# Microplastics, potential threat to patients with lung diseases

**DOI:** 10.3389/ftox.2022.958414

**Published:** 2022-09-28

**Authors:** Kuo Lu, Danting Zhan, Yingying Fang, Lei Li, Guobing Chen, Shanze Chen, Lingwei Wang

**Affiliations:** ^1^ The Department of Respiratory Diseases and Critic Care Unit, Shenzhen Institute of Respiratory Disease, Shenzhen Key Laboratory of Respiratory Disease, The Second Clinical Medical College of Jinan University (Shenzhen People’s Hospital), Post-Doctoral Scientific Research Station of Basic Medicine, Jinan University, Guangzhou, China; ^2^ Guangdong Provincial Key Laboratory of Brain Connectome and Behavior, CAS Key Laboratory of Brain Connectom aend Manipulation, The Brain Cogntion and Brain Disease Institute (BCBDI), Shenzhen Institutes of Adavnced Technology, Chinese Academy of Sciences, Shenzhen-Hong Kong Institute of Brain Science-Shnezhen Fundamental Research Institutions, Shenzhen, China; ^3^ Department of Microbiology and Immunology, Institute of Geriatric Immunology, School of Medicine, Jinan University, Guangzhou, China

**Keywords:** microplastics, lung disease, epithelial barrier dysfunction, inflammatory response, redox imbalance

## Abstract

Air pollution is one of the major risk factors for lung disease. Microplastics are a ubiquitous environmental pollutant, both indoors and in outdoor air. Microplastics have also been found in human lung tissue and sputum. However, there is a paucity of information on the effects and mechanisms of microplastics on lung disease. In this mini-review, we reviewed the possible mechanisms by which air microplastics’ exposure affects lung disease and, at the same time, pointed out the limitations of current studies.

## Introduction

Microplastics are air pollutants that have attracted intense attention in recent years. Microplastics have been detected in the air of large, densely populated cities (e.g., Beijing, Shanghai, London, etc.) (Liu et al., 2019; Li et al., 2020; Wright et al., 2020), and the type and concentration of airborne microplastics are influenced by community lifestyles, anthropogenic activities, and meteorological conditions (Chen et al., 2020; Mbachu et al., 2020). Atmospheric microplastics can settle on the ground or float by wind and air movement. Due to the small size, airborne microplastics can be directly inhaled by humans (Kaya et al., 2018; Chen et al., 2020). Microplastics are present in indoor and outdoor air, although the concentration of microplastics in the indoor environment is greater than that in the outdoor environment. Indoor objects, materials, and furnishings produce plastic debris due to abrasion, and microplastics from these sources have a much greater impact on humans than the polymers contained in food and beverages (Rist et al., 2018). Inhalation of plastic fibers and particles, especially by exposed workers, often leads to respiratory discomfort due to inflammatory responses in the airways and interstitium. Even at very low ambient concentrations, the susceptible individuals are at risk of developing lesions (Atis et al., 2005; Prata, 2018).

**FIGURE 1:**
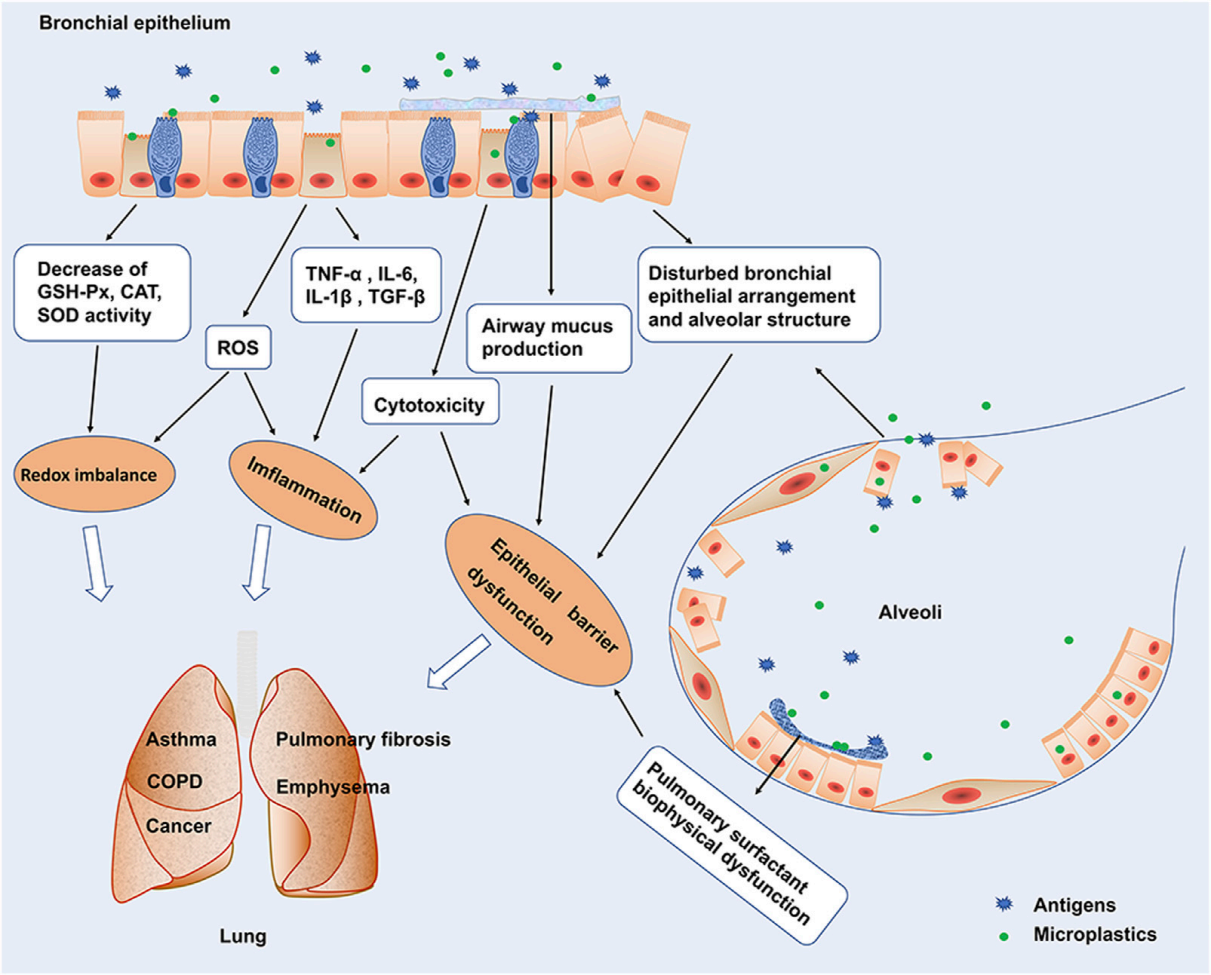
Potential effects and possible mechanisms of microplastics on respiratory. Microplastic exposure interferes with the biophysical function of pulmonary surfactants and disrupts alveolar structure and airway barrier function. Exposure of microplastics to lung epithelial cells inhibits epithelial cell proliferation and induces epithelial cell apoptosis. Microplastics stimulates the production of pro-inflammatory cytokines such as TNF-α, IL-6, IL-1β production, and TGF-β production by lung epithelial cells. In addition, exposure to microplastics caused a significant decrease in oxidative stress-related GSH-Px, CAT, and SOD activities, induced ROS production, and Redox imbalance.

The deposition of microplastics in the human respiratory system has been demonstrated. The presence of microplastics has been found in the human lung (Amato-Lourenco et al., 2021; Jenner et al., 2022) and in the sputum of patients with respiratory diseases (Huang et al., 2022). A maximum of 565 particles/10 ml and 21 types of microplastics were identified (polyurethane, polyester, chlorinated polyethylene, and alkyd varnish, accounting for 78.36% of the total microplastics) in sputum. Microplastics are inhaled into the respiratory tract and can be excreted with sputum, and personal smoking habits and invasive tracheal examinations have been associated with microplastic exposure (Huang et al., 2022). However, studies on the effects of these microplastics deposited in the lungs, especially in patients with respiratory diseases, are still in the initial stage. Here, we present an overview of the potential effects and possible mechanisms of microplastics on respiratory health (Figure 1) with the aim of providing an awakening to the detriments of microplastics.

## Possible mechanisms by which microplastics affect respiratory health

### Epithelial barrier dysfunction

On the alveolar surface, there is a complex layer of surface-active substances consisting of proteins and lipids. These pulmonary surfactants form a complex structural fluid-fluid interface and form a stable film in the alveoli, which has the effect of reducing surface tension, preventing alveolar collapse during exhalation, and reducing the energy consumed by respiration. In addition, pulmonary surfactants have important transport properties in the lung. The pulmonary surfactant flows up the terminal airways and reduces the formation of liquid plugs that can obstruct terminal airways at end-expiration (Hermans et al., 2015). Further up the bronchial tree, the mixing of pulmonary surfactant with airway mucus improves the mobility and clearance of mucus by bronchial epithelial cilia. Pulmonary surfactant flowing up the airway tree helps to remove particulates and pathogens inhaled from the alveoli and distal airways (Podgorski and Gradon, 1990). Thus, disruption of surface-active substances reduces lung defenses. Nanoplastics are transferred to the alveolar air-water interface by vesicle fusion and cause biophysical dysfunction of pulmonary surfactant by disrupting its ultrastructure and mobility, leading to collapse of the pulmonary surfactant film (Li L. et al., 2022a). Polystyrene microplastics (PS-NPs) adsorb more phospholipid components of pulmonary surfactant than that of proteins. Polystyrene accelerates the conversion between ascorbic acid and deoxyascorbic acid, generates hydrogen peroxide (HOOH) in the simulated lung fluid, and leads to an increase in hydroxyl radical (-OH) content, which ultimately alters the phase behavior, surface tension, and membrane structure of pulmonary surfactant (Shi et al., 2022). The change in surface tension in turn affects the migration of nanoparticles and the collapse of the surfactant film. Furthermore, *in vitro* experimental studies revealed that PS-NPs reduce trans-epithelial resistance by depleting tight junction proteins. Expression of cellular matrix metallopeptidase 9 and surface-active protein A were elevated after PS-NPs treatment, suggesting that PS-NPs exposure may reduce lung repair capacity and cause lung injury (Yang et al., 2021). The disruption of epithelial barrier function makes it easier for foreign substances, such as allergens and toxins, to enter the interstitium and bloodstream, which may lead to the development of lung diseases such as asthma and chronic obstructive pulmonary disease (COPD) (Zhao et al., 2020).

### Cytotoxicity

Pulmonary epithelial cells are located at the interface between the host and the environment, where they are directly exposed to inhaled air as well as to airborne microplastics. Microplastics are able to internalize into epithelial cells, and the rate of internalization is influenced by physicochemical properties such as particle size and surface charge. Microplastics with small particle size are internalized more rapidly than larger particles. The internalization and intracellular accumulation of microplastics with positive surface charge is greater than that of microplastics with negative surface charge. *In vitro* experiments showed that internalization of microplastics led to blebbing morphology and activation of apoptotic signals in alveolar epithelial cells under cyclic stretches (Yang et al., 2021). After giving nasal microplastic drops (1–5 μm, 300 µg/20 µl) to mice, microplastics were found in the airways, alveoli, and interstitium, indicating that microplastics can penetrate the alveolar epithelial barrier (Lu et al., 2021). Pathological examination of rats with tracheal injection of microplastics (a maximum concentration of 2 mg/200 μl) showed disruption of alveolar structure and disorganized arrangement of bronchial epithelium (Fan et al., 2022). These studies suggest that microplastic exposure will cause lung injury. Therefore, prolonged exposure to microplastics may lead to the development and progression of lung diseases.

The effect of internalization of microplastics on cell viability is still under discussion. Goodman et al. (2021) reported that treatment with high concentrations (100 μg/ml) of microplastics did not exhibit toxicity to human alveolar A549 cells, with cell survival rates above 93%. However, microplastics inhibit cell proliferation and cause significant changes in cell morphology. However, Yang et al. (2021) evaluated the relationship between polystyrene microplastics and lung injury using two types of human lung epithelial cells (BEAS-2B) and human alveolar epithelial cells (HPAEpiC) and noted that polystyrene microplastics significantly reduced cell viability in a dose-dependent manner (Yang et al., 2021). Pro-apoptotic proteins such as A549DR5, caspase-3, caspase-8, caspase-9, and cytochrome c were significantly upregulated in alveolar epithelial cells after treatment with polystyrene nanomicroplastics, suggesting that PS-NPs can induce apoptosis (Xu et al., 2019). This may be related to the chemical nature of microplastics, surface charge, concentration, exposure time, photoaging of the surface, and whether other toxic substances are adsorbed on the surface. For example, a study by Kecheng et al. opined that fresh Phenol-formaldehyde resin microplastic (PF-MP) treatment had no significant effect on cell viability, but photo-aged PF-MP was able to significantly reduce cell survival (Zhu et al., 2020).

### Inflammatory response and redox imbalance

Rather than acting as a simple physical barrier, the airway epithelium detects allergens, microplastics, and other irritants and then helps to organize the subsequent immune response by releasing a large number of secretory signals. Microplastics’ exposure induces epithelial cell inflammation. The pro-inflammatory cytokine TNF-α may have a momentous impact on microplastics-induced airway inflammation. Transtracheal injection of polystyrene microplastics increased the expression of the pro-inflammatory cytokines TNF-α, IL-6, and IL-1β in alveolar lavage fluid in rats (Fan et al., 2022). Lu et al. (2021) observed that microplastic exposure increased the production of TNF-α in BALF of mouse. Expression of inflammatory proteins (TGF-β and TNF-α) in the lung tissue of rats exposed to polystyrene microplastics increased in a concentration-dependent manner (Lim et al., 2021). In *in vitro* experiments, PS-NPs induced significant increases in pro-inflammatory cytokines such as IL-8, NF-κβ, and TNF-α (Xu et al., 2019). TNF-α is closely associated with dysregulated airway inflammation in asthma. Whereas TNF-α is involved in the interaction between mast cells and smooth muscle cells and might be closely related to the development of airway hyperresponsiveness in asthmatic patients. In addition, TNF-α has the ability to promote neutrophil recruitment, induction of glucocorticoid resistance, myocyte proliferation, and stimulation of fibroblast growth and maturation into myofibroblasts by promoting the expression of TNF-α (Berry et al., 2007). These are all latent mechanisms that are associated with refractory asthma. The above results suggest that microplastics can affect and would be harmful to asthma patients. Moreover, TGF-β is also involved in pulmonary diseases. For instance, TGF-β regulates a variety of cellular processes such as epithelial cell growth inhibition, alveolar epithelial cell differentiation, fibroblast activation, and extracellular matrix organization, which are associated with tissue remodeling in pulmonary fibrosis and emphysema. Upregulation of TGF-β is seen in major lung diseases such as pulmonary fibrosis, emphysema, bronchial asthma, and lung cancer (Saito et al., 2018). Collectively, overexpression of TGF-β induced by microplastics may engage in the course of these respiratory diseases.

Redox imbalance may be an additional mechanism by which microplastics induce effects on chronic respiratory diseases. Oxidative stress caused by redox imbalance is one of the mechanisms contributing to the pathogenesis of several lung diseases such as asthma, COPD, and acute respiratory distress syndrome (ARDS) (Hecker, 2018). Exposure to microplastics isolated from the environment showed a depletion of antioxidants in simulated lung fluid (Abbasi et al., 2019), and microplastics’ exposure also result in increased ROS production in mouse lung tissue. Inflammation induced by ROS is the initial stage leading to tissue damage. What’s more, microplastic exposure alters the oxidative status and antioxidant capacity of bronchial epithelial cells. GSH-Px, CAT, and SOD activities associated with oxidative stress are significantly reduced by microplastics in a dose-dependent manner, resulting in excessive oxidative stress (Li Z. et al., 2022b). Both inflammation and redox imbalance are closely related to pathophysiological processes; therefore, the potential threat of inhaled microplastics should be given adequate attention.

### Synergistic effects with allergens

As aforementioned, microplastics increase the permeability of allergens by disrupting alveolar barrier function. Our previous study found that co-exposure to microplastics (300 µg in 20 µl saline) and HDM (house dust mite) increased airway mucus secretion and aggravated HDM-induced airway inflammation in asthmatic mice. In addition, co-exposure to microplastics and HDM induced increased expression of MALT1 in lung tissue of mice (Lu et al., 2021). MALT1 is widely expressed in lymphoid, mast, and endothelial cells, acting as a scaffolding protein and as a substrate-specific protease. MALT1 protease has bifacial roles in the allergic disease by mediating IgE-dependent mast cell cytokine production and histamine-induced endothelial permeability (Alfano et al., 2020). This suggests that co-exposure to microplastics and allergens may synergistically exacerbate allergic diseases.

## Limitations

In studies investigating the side effects of microplastics, microplastics of different chemical materials, particle sizes, or concentrations have been used. Among them, polystyrene microplastics are the most used. Polystyrene microplastics are indeed one of the major air pollutants; however, they do not exist alone in the real environment. In the air, microplastics are present together with other pollutants and even have organic substances or metal ions adsorbed on their surfaces. Microplastics and these substances cause a synergistic effect in terms of side effects on humans. Therefore, in-depth studies are needed to investigate the effects of mixed exposure to multiple microplastics on respiratory health. Secondly, microplastics can release harmful chemicals in the natural environment or even in the physiological environment; whether it is these chemical hazards or the particles themselves that have greater health side effects has not been reported. Furthermore, except in some occupational settings, the concentration of microplastics in the air is very low, but long-term exposure, and the relationship between such small doses of long-term microplastics exposure and respiratory disease, needs more clinical observational studies and long-period animal model experiments. Finally, it has been found that nano-microplastics section inhaled *via* airways can be transferred to other organs *via* blood circulation; what are the effects of these microplastics transferred to other organs on these organs? Whether microplastics deposited in the lungs and other organs can be removed from the body, how they are removed, and how long it takes to do so are all issues that need to be further explored.

In conclusion, although research on the respiratory health effects of microplastics remains scarce, the negative effects that microplastics can have on respiratory health are clearly evident. This review provides an overview of the possible threats and mechanisms of respiratory health effects of airborne microplastics and points out the limitations of current research. Meanwhile, we herein call for more attention to be given to microplastics and the respiratory health threats it poses.
