# Erratum: Microplastics, potential threat to patients with lung diseases

**DOI:** 10.3389/ftox.2022.1119994

**Published:** 2022-12-20

**Authors:** Frontiers Production Office

**Affiliations:** Frontiers Media SA, Lausanne, Switzerland

**Keywords:** microplastics, lung disease, epithelial barrier dysfunction, inflammatory response, redox imbalance

Due to a production error, a spelling error was made in the **article title**: “Micropalstics, potential threat to patients with lung diseases” instead of “Microplastics, potential threat to patients with lung diseases”.

The publisher apologizes for this mistake. The original version of this article has been updated.

